# Genomics Education in the Era of Personal Genomics: Academic, Professional, and Public Considerations

**DOI:** 10.3390/ijms21030768

**Published:** 2020-01-24

**Authors:** Kiara V. Whitley, Josie A. Tueller, K. Scott Weber

**Affiliations:** Department of Microbiology and Molecular Biology, 4007 Life Sciences Building, 701 East University Parkway, Brigham Young University, Provo, UT 84602, USA; kiara.vaden@gmail.com (K.V.W.); josieat@gmail.com (J.A.T.)

**Keywords:** personal genomics, Human Genome, sequencing, genetic testing, bioethics, genomics education, science education

## Abstract

Since the completion of the Human Genome Project in 2003, genomic sequencing has become a prominent tool used by diverse disciplines in modern science. In the past 20 years, the cost of genomic sequencing has decreased exponentially, making it affordable and accessible. Bioinformatic and biological studies have produced significant scientific breakthroughs using the wealth of genomic information now available. Alongside the scientific benefit of genomics, companies offer direct-to-consumer genetic testing which provide health, trait, and ancestry information to the public. A key area that must be addressed is education about what conclusions can be made from this genomic information and integrating genomic education with foundational genetic principles already taught in academic settings. The promise of personal genomics providing disease treatment is exciting, but many challenges remain to validate genomic predictions and diagnostic correlations. Ethical and societal concerns must also be addressed regarding how personal genomic information is used. This genomics revolution provides a powerful opportunity to educate students, clinicians, and the public on scientific and ethical issues in a personal way to increase learning. In this review, we discuss the influence of personal genomics in society and focus on the importance and benefits of genomics education in the classroom, clinics, and the public and explore the potential consequences of personal genomic education.

## 1. Introduction

Genomics has become a central pillar driving modern scientific research and discovery. Beginning with the Human Genome Project that was initiated in 1990 and completed in 2003, the study of genomics has rapidly evolved in the last 30 years. Today, it is possible to rapidly sequence an organism’s genome and determine critical insights into many areas including health, ancestry, and traits [1]. The vast amount of genomic information obtained over the last decade has provided crucial insights into various health issues, which have significantly improved diagnosis and treatment for many diseases [2]. For example, personal genomics data is now used to distinguish different cancers, such as Burkitt’s lymphoma and diffuse B-cell lymphoma, and enables prediction of cancer sensitivity to drugs and treatment selection [3]. Additionally, genomics data has been used in the clinic to help monitor the likelihood of graft rejection by measuring gene expression in peripheral lymphocytes [4]. Important advancements in genomics have expanded to other organisms, such as bacteriophages which are currently being characterized and developed for agricultural treatments such as fire blight [5]. The use of genomics to characterize bacteriophages allows their genomes to be analyzed for bacterial specificity and to identify genes that may be beneficial or harmful in targeting specific bacterial populations [6]. In addition, genomics has played a key role in ushering in new fields of research such as the microbiome, providing many important basic science insights as well as potential treatments for many diseases [7]. For example, patients with *Clostridium difficile* infection have significantly benefited from microbiome transplants from healthy donors, thereby providing an effective treatment for a highly morbid infection [8]. Genomics provides information regarding the healthy donor’s microbiome profile for healthcare providers to know the characteristics and composition of the transplant bacterial community [9]. These rapid advancements have been enabled by the arrival of next-generation sequencing in 2006, which inspired a technological wave of new methods and applications that have revolutionized DNA sequencing [1]. With many high-throughput sequencing methods now readily available, the cost and time to obtain genomic data has decreased significantly. The Human Genome Project took 13 years and cost $95,263,072, whereas today some companies charge less than $1000 to sequence your entire genome in 24 hours [10]. 

Despite the exciting technical advances in genomics, many societal, ethical, and scientific concerns remain. The significant decrease in cost has made genomic sequencing more accessible to businesses outside of academic and clinical research, leading to the development of direct-to-consumer genomic profiling [11]. Some studies have shown that direct-to-consumer genomic profiling has been beneficial in identifying and preventing disease [12]. However, many health care professionals remain concerned about direct-to-consumer genomic profiling, as results may lack clinical validity, can be misinterpreted by patients, and can psychologically impact some patients’ well-being [11,13,14]. For these reasons, genetic counselors are trained to interpret results and provide education to patients to help them make informed healthcare decisions, yet many people are unlikely to utilize genetic counselors and opt to interpret their results on their own or see a physician instead [15]. However, many healthcare professionals that are not specialized in genomics, such as primary care physicians, feel unprepared to answer patient questions about their genomic profile [16,17,18]. This rapidly moving field is uncovering societal challenges in how to properly incorporate and utilize genomics as a part of our understanding of health and disease and medical practice.

The dramatic increase of public interest for genomic profiling from 29% to 37% between 2008 to 2011 also presents potential ethical issues [19]. The Genetic Information Nondiscrimination Act of 2008 (GINA) prevents employment and health insurance discrimination based on genetic information; however, ethical and privacy concerns remain, primarily regarding data access [14]. Recently, law enforcement and public attention has focused on using genealogical genomic profiling to find relatives and ancestors via direct-to-consumer genetic testing [20]. Famously, the Golden State Killer was recently apprehended after police used genomic information obtained through GEDmatch, a genealogy company, to trace the killer through familial genomic profiling [21]. Currently, the Federal Bureau of Investigation is addressing guidelines for using genealogy, stating that investigations only utilize this source of information if CODIS reveals no matches and only using public databases which inform the users of this possible forensic use [22]. More recently, the Pentagon advised all military personnel against direct-to-consumer genetic testing [23]. The GINA does not apply to military personnel, therefore, the discovery of unknown genetic markers or inaccurate results which affect military physical requirements could jeopardize the member’s service, as well as potentially affect military security through exposure of genetic information [24]. While personal genomics is a valuable tool for police to identify and locate suspects, it raises privacy concerns for the public and military and requires public discussion and education about the use of genomic information [25].

As genomics remains an emerging discipline, much work still needs to be accomplished for the genomic therapeutic potential of precision medicine to be fully realized. Most genome-wide association studies (GWAS) have data from subjects of European descent, limiting the interpretation of and increasing uncertainty of disease risk for non-European subjects [26]. Cancer biomarkers such as BRCA1/2 are significant indicators of breast cancer; however, many BRCA1/2 mutations and unknown genetic variants are poorly defined, making disease risk uncertain and disease assessment and diagnosis complicated [2,27]. Unfortunately, these complications in data interpretation create considerable issues, such as significant risk of misdiagnosis, psychological impacts on patient and relatives, unnecessary medical procedures, and decreased confidence in proper diagnosis and treatment [28]. Consequently, a lack of genomic understanding regarding how to interpret genomic test results is an important need for genomic education about what can and cannot be concluded from personal genomic information. As genomics is a critical branch of genetics, it is equally important that a concrete understanding of genetics preludes and accompanies genomic education. Beyond its potential in ancestry identification, law enforcement, and health care, this personal genomics revolution provides a powerful opportunity to educate students, clinicians, and the public on relevant scientific and ethical issues. Effective genomic education enables healthcare professionals, students, and the general public to understand the benefits of genomic discoveries with important applications in ancestry, health, traits, and forensics [29,30,31]. In this review, we discuss recent literature on personal genomics education in academic, professional, and public settings and examine the benefits for enhanced student learning in the classroom and potential consequences of personal genomic education.

## 2. Academic Genomics Education

### 2.1. High School Education

High school is one of the earliest academic institutions where students are introduced to genomics. As genomic information becomes more common, it is imperative that molecular and genomics education begin early to not only provide background to future health professionals, but to familiarize all citizens with the limits and possibilities of genomic information in healthcare and beyond [32]. Genomic education in high school is also important because high school biology is frequently not only the initial, but also the final formal exposure to genomics for many Americans [33]. Many high school students have the interest, maturity, and intelligence to learn and understand genomics, yet few studies involving high school students and genomic education have been conducted in the past five years (Table 1) [34,35,36,37,38,39,40,41,42,43,44].

Each of these studies incorporated teaching genomic principles to high school students. Of the nine studies identified, three utilized genomic online tools to teach concepts [36,37,39]. Many of the tools utilized in these studies, such as the NCBI database and next generation sequencing, are critical for genomic studies in college. Utilizing these technologies in high school classrooms can facilitate interest in and preparation for careers focused on genomics [34].

One interesting study utilized media representation of genomics to teach students to think critically of media portrayal and framing of science topics [44]. With media being readily available, it is essential for students to develop critical thinking and reasoning skills so they can identify misconceptions in the media regarding science topics [45]. Teaching genomics to high school students can also help public perception and understanding [38]. Athanasiadis and colleagues held an educational symposium for students and teachers across Denmark which garnered positive media attention as the symposium was extensively broadcast on primetime news and published online. These studies demonstrate the importance and influence of media in teaching genomics.

Of the nine studies described in Table 1, seven studies surveyed student learning (indicated by asterisk next to paper title in Table 1). Of the seven studies, six of the studies demonstrated that student knowledge and interest increased as the model was implemented, indicating these learning models have the potential to improve genomics education. Students felt that learning genomics helped prepare them for future academic study [37] and created a more positive learning experience [40]. Thus, these publications indicate that incorporation of genomic education can be beneficial for high school student learning [38,39,44].

It is also important to recognize the need for teacher education and curriculum development specific for this new field of genomics. Without training and updated curriculum, teachers can be uncomfortable or hesitant to implement new teaching techniques. Programs such as Teaching the Genome Generation (TtGG) are enabling teachers to become more confident in genomic education as the programs teaches them hands-on laboratory skills that they can use to teach students real-life case scenarios [41]. It is interesting to note that teaching style can also impact learning experiences [40]. A recent study identified the importance of assessing student knowledge and tailoring lessons based on student knowledge gaps in order to positively deepen student knowledge and attitudes towards genomic information [44]. By helping to open educational doors for teachers, they can be better prepared and equipped to engage their students in learning genomic principles. Programs like TtGG can also provide collaboration opportunities for teachers to discuss and potentially improve genomic curriculum [41]. A recent study found that of 11 selected textbooks, 73.8% of the genomic content only discussed classic single gene diseases, such as sickle cell anemia, indicating that many high school textbooks likely need updating to include multifactorial or complex conditions such as cancer or diabetes [33]. Understanding that many common diseases are controlled by numerous factors, including genetics and the environment, could be pivotal for preparing students with knowledge needed for a future filled with genomic applications [33]. Thus, these studies indicate that not only teaching genomics to high school students is important but educating teachers and modernizing curriculum is also vital to facilitate student learning.

### 2.2. Undergraduate and Graduate Education

**Figure 1:**
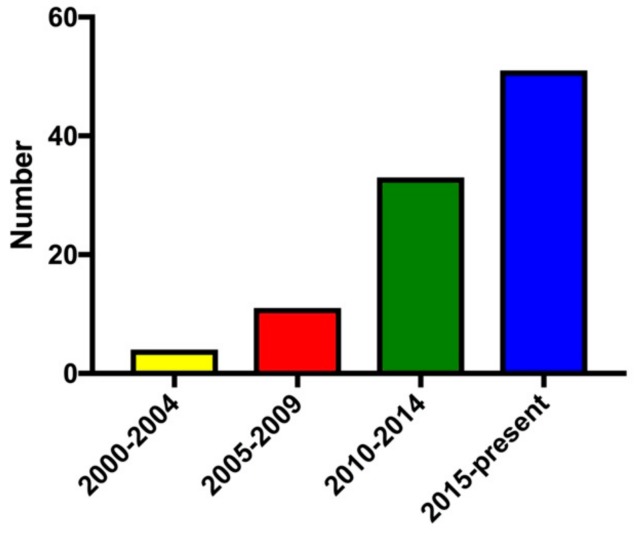
Number of college genomic education publications since 2000: Ninety-nine studies from 2000 to 2019 were surveyed. Of the 99 published studies, 4 were published between 2000 and 2004, 11 were published between 2005 and 2009, 33 were published between 2010 and 2014, and 55 were published in the last five years (2015 to present).

Teaching undergraduate and graduate students is another key academic focal point of genomic education. Over the last twenty years, studies involving genomic education of college students has steadily increased (Figure 1). Of the 99 personal genomics studies we identified in the past 19 years, nearly half of the studies were published in the last four years, indicating that genomic education is being implemented at an increased rate in the college setting. Interestingly, many studies performed earlier in the decade focused on teaching upper-level junior and senior life science majors [46,47], but more recently introductory biology courses have begun to incorporate genomics into their class curriculum [48,49].

**Figure 2:**
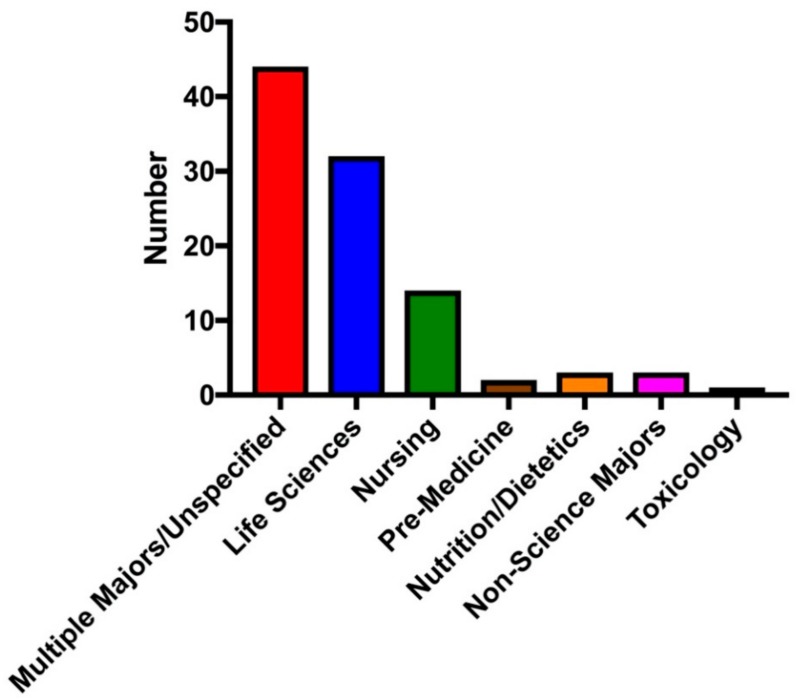
Targeted or reported college student major composition of undergraduate/graduate classes. Of the 99 personal genomics studies we surveyed, we analyzed the target student audience or the reported class major composition. We found that 44 of the studies spanned multiple majors or did not specify their target student audience, 32 of the studies were focused on life science students, 15 on nursing students, 2 on pre-medicine students, 3 on non-science majors, and 4 on other disciplines.

College genomic education is also targeting a variety of student majors (Figure 2). Many of the studies we surveyed did not specify their class’s major composition or the class composition spanned multiple disciplines [50]; however, 32 of the studies focused on the genomic education of life sciences majors, primarily focusing on biology and biochemistry majors [51,52]. It is immensely important that genomics education be emphasized for life science majors, as many of them will pursue healthcare careers and play an important role in disseminating genomic information to their patients [53]. Surprisingly, only a few studies focused specifically on non-science majors. It is possible that studies we surveyed that did not specify their class target audience or composition had an impact on non-science majors taking the class. Educating non-science majors in genomics will be an important avenue to educate students who do not specialize in a science major and prepare them to understand and recognize the importance and influence of genomics in society [54].

Remarkably, 15 of the studies were focused on examining genomic education for nursing students. According to many studies, there is an increasing need for nursing students to be educated in genomics as their knowledge of and attitude toward genomics is poor, and many do not feel prepared to use genomics in the clinic [55,56,57]. Nurses are also crucial healthcare providers that, alongside physicians, are part of the frontline in providing genomic education to patients [58]. Studies have shown that providing genomic courses for nursing students improves student confidence and genomic literacy [59,60]. Nurses are a critical part of the healthcare workforce, and therefore it is important for nursing classes to incorporate genomics into their curriculum [59].

Of the studies surveyed, there were notable learning models and resources that have been successfully used to teach college-level genomics, particularly to life sciences majors. Course-based undergraduate research experiences (CUREs) are a major method of undergraduate genomic education [61]. CUREs are designed to provide learning and hands-on research experience to undergraduate students [62]. In surveying the literature, three studies about genomic CUREs stood out. Olson et al. published a genomic CURE designed to screen gene expression in Drosophila [63]. With over 250 co-authors, this study demonstrated significant success in involving a large introductory class size in research-based genomic learning. This study also positively impacted student learning and significantly increased the number of undergraduate students that continued to pursue a STEM degree. Reeves et al. studied the conservation of the methionine pathway in yeast to teach genomics to students in a CURE-based setting [51]. Five semesters of teaching this course revealed that many students significantly increased their knowledge base and research skills. Importantly, underrepresented minority students had larger knowledge gains than other groups, indicating a positive movement toward cultivating successful professionals to bring diversity to their field [64]. Bhatt and Challa designed an introductory course which taught genomic principles through hands-on experience with CRISPR-Cas9, which has become one of the most important genome-editing tools in modern science [65]. The study utilized zebrafish to analyze genetic mutations with their corresponding phenotype and allowed students to design and test CRISPR-Cas9 templates in order to disrupt certain zebrafish genes. Additionally, this CURE demonstrated student knowledge increases, and students also reported that they gained transferrable skills and insights applicable outside of the CURE. Given these observations, CUREs are a vital educational means for teaching genomics to college students.

One of the most widespread CURE programs is the Genomics Education Partnership (GEP). The GEP consists of over one hundred universities who are collaborating to bring genomic learning to their classrooms through research-based methods. Students who participate in GEP projects actively learn how to perform genome annotation of Drosophila and produce gene profiles of specific portions of different Drosophila genes [66]. Students who have worked on GEP projects have reported increased knowledge gains and value their learning experience and contribution to science [67]. GEP projects are also very accessible to most universities as only computers are needed and are versatile since projects can be short and be accomplished by many students in comparison to a typical research-based class [66]. These studies have demonstrated that GEP is a valuable teaching resource for undergraduate genomic education [67].

Science Education Alliance Phage Hunters Advancing Genomics and Evolutionary Science (SEA-PHAGES), also called Phage Hunters, is another CURE program which has been instrumental in teaching undergraduates about genomics [68]. Phage Hunters was designed to be a year-long course for beginning undergraduates where bacteriophages are isolated from soil samples, sequenced, and characterized the following semester for putative gene function [69]. Seventy-three universities have implemented beginning courses of Phage Hunters and have demonstrated that Phage Hunters increases student learning, interest, and pursuit of academic education in STEM majors [49,69,70]. Some universities have modified the Phage Hunters course so that the second semester of gene characterization is included into other biology classes and have demonstrated equal academic value and success [49]. In addition to the benefits of student learning, students often become authors on scientific publications of bacteriophage characterization, thereby improving their academic portfolio as well [6]. These studies demonstrate that Phage Hunters is a valuable CURE which can be implemented in large undergraduate classes and be advantageous for teaching genomics.

A valuable way to teach genomics to college students and significantly improve interest and engagement is to personalize the data they are analyzing. We highlight two genomics education studies performed at Brigham Young University. The first study examined the effects of giving students personal genomics kits and found that just the anticipation of getting personal data in the future improved student learning and interest in their related course material as compared with students who were analyzing genomics data from an unidentified individual [46]. Students who were going to receive personal genomic results spent more time studying for their lecture-based molecular biology, genomics, or immunology courses, which increased their confidence to interpret the results they would receive as well as better understand the risks and benefits of using direct-to-consumer genetic testing. The second study examined the effects of giving students microbiome kits and found that students who analyzed their personal microbiome were more engaged in learning and had a more positive attitude towards the class as a whole as compared with students analyzing microbiome data from an unidentified individual [47]. Students who analyzed their own microbiome data also reported they visited more websites and sources to learn about the microbiome, indicating that receiving personal microbiome kits increased their interest in learning. Additionally, students who received microbiome kits felt the course was more applicable to them than those who analyzed microbiome data from an unidentified individual. While these studies did not directly involve students performing research like in CUREs, it is important to note that students analyzing personal genomics or microbiome data spent more time studying the topic and their interest increased in comparison to control groups who only received data from an unidentified individual [46,47]. These studies indicate that providing genomics data that is personal to you is a valuable tool for increasing student interest and engagement and educating undergraduate and graduate students in genomics topics.

### 2.3. Medical School Education

Genomics is becoming an integral part of medicine and physicians must be prepared to understand and communicate complex genomic information to patients and the public in a simple and accurate manner [71]. This critical need for genomics in medical school has already been incorporated into many medical student training programs [72]. The Association of Professors of Human and Medical Genetics has developed a core curriculum for medical students, which focuses on understanding genomic variance, disease phenotype, genomic technologies, and direct-to-consumer testing [72]. Despite this, there has been debate about the most effective educational models with which to teach genomics to medical students [73]. Use of personal genomics in medical school has had mixed results. One study found that student interest did not increase and that many students did not think personal genomics was useful [74]. In contrast, another study found that genomics enhanced learning and provided a positive learning experience [75]. Interestingly, the difference between these two studies was the addition of personal genomic testing to the genomics course. Students who did personal genomic testing had significantly higher test scores and self-reported that their conceptual knowledge in genomics had significantly increased [75]. These studies suggest that including personal genomics testing into medical school classrooms may be beneficial in increasing student knowledge and interest about genomics.

**Figure 3:**
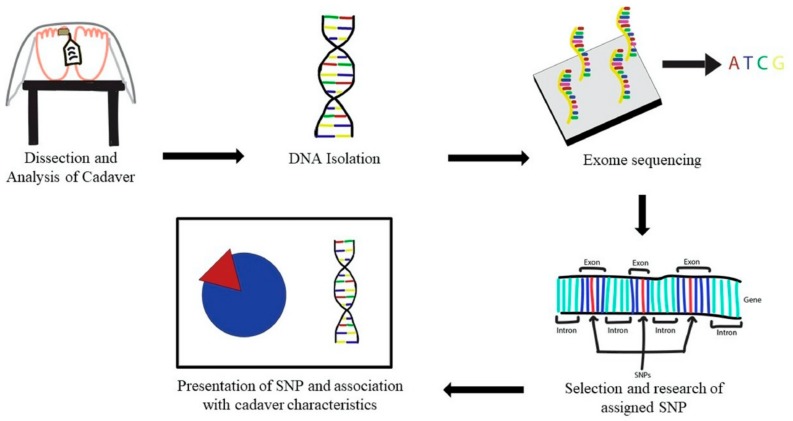
Workflow of first-year medical anatomy lab and genomics. Cadavers are dissected by students and traits about the cadaver are observed and recorded. DNA samples are isolated from various organs, including the heart and liver. Samples are sequenced and professors assign SNPs to different student groups who characterize the SNPs and associate them with cadaver traits. The students present their research in a 15-minute PowerPoint presentation [76].

The Anatomy to Genomics (ATG) Start Genetics medical school initiative is another promising method for teaching medical students about genomics [76]. The ATG initiative incorporates first-year anatomy with genomics to teach students about genomic sequencing application and strengthen their understanding in anatomy. As part of the initial study at Lewis Katz School of Medicine at Temple University, DNA samples were taken from the liver, skin, cardiac, and skeletal tissue. Seven different cadavers were dissected, DNA samples were analyzed, and students were asked to research single nucleotide polymorphisms (SNPs) (Figure 3). This ATG program allowed students to draw conclusions about the cadaver’s traits and disease phenotype regarding its genomic profile. Another group performed a similar study on a cadaver who had been diagnosed with idiopathic pulmonary fibrosis [77]. Interestingly, the genomic characterization of the cadaver revealed that the individual could have died from nonspecific interstitial pneumonia rather than idiopathic pulmonary fibrosis as had been suspected by the attending physician. The students found that the cadaver had a SNP (rs35705950) in the promotor region of the mucin glycoprotein MUC5B, which is associated with risk of developing idiopathic pulmonary fibrosis, but the SNP had never been linked to nonspecific interstitial pneumonia. These findings confirm that the SNP in MUC5B promotor is related to the development of lung disease and that the cadaver was genetically predisposed to lung disease. Together, these studies demonstrate that a combined anatomy and genomics approach is advantageous to student learning and furthering clinical research.

### 2.4. Education for Genetic Counselor Students

The need for genetic counselors has never been more important with the rise of genomic technologies and healthcare services available to the public [78]. There has been a significant increase in demand for genetic counselors due to the genomics revolution; however, lack of funding, counseling supervision, and training slots has created a nationwide shortage of genetic counselors [79]. Genetic counseling programs are designed to educate students to fill a professional role in providing genomic-related medical advice to patients and medical practitioners [80]. With specialized training and certification, it is extremely important that up-to-date genomics and counseling education be emphasized in genetic counseling training programs [81]. Genetic counseling instructors were surveyed about the incorporation of genomics into genetic counseling training and found that the majority felt genomics was important to include and that most topics were currently included or being established [82].

Some institutions such as Stanford University are focusing on experiential learning utilizing “rotations” for genetic counseling students to teach variant interpretation using a case-based system [83]. Three distinct “rotations” were created, focusing on different settings of genetic counselors, i.e., clinical, research, or laboratory-based rotations. Students reported that these rotations were particularly useful in preparing them for their chosen discipline. Another study conducted at the University of North Carolina at Greensboro and Duke University found that teaching clinical application of genomics testing should be emphasized [81]. Students in this study reported that while genomics testing curriculum prepared them to pass the ABGC board exam, 50% of students felt unprepared to fill the role of providing genomic testing interpretation on a clinical scale. These studies emphasize the need to analyze genetic counseling student training and perhaps incorporate more access to clinical or hands-on experiences to improve genetic counselor preparation [81,83].

## 3. Genomics Education for Clinical Professionals and the Public

Advancements in genomic education on the academic level have rapidly expanded over the past decade; however, educating healthcare professionals that are currently practicing and the public is equally critical and must be addressed [84]. Current health professionals need to be educated as they administer developing genomic technologies in the healthcare industry as they may have been trained before the genomics era, and the public needs to be educated as the influence of genomics is becoming more personal and frequent [50,85]. Here we will discuss recent strategies and challenges in the education of practicing nurses, practicing physicians, and the public.

### 3.1. Nurse Genomic Education

Nurses are a pivotal part of the healthcare workforce in implementing genomics into everyday patient care as they lead changes in healthcare and ethical practices and interact frequently with patients [86]. However, many nurses lack understanding of genomic principles, resulting in healthcare deficits that can be improved through genomic technologies [87]. Eighteen countries were recently surveyed on existing genomic education and three countries (United Kingdom, Japan, and the United States) have genomics integrated into nursing student classes and competencies, but only Israel requires all practicing nurses to take a mandatory 28 h course in order to remain certified to practice [58]. Other studies have measured genomic competence, knowledge, and attitudes and found that genomic integration into nursing practice is currently lacking, but education programs improved competency [88]. These studies indicate genomic competency desperately needs to be incorporated into continued nursing education.

Nursing professionals have begun developing initiatives to address genomic educational needs. In 2012, the Genomic Nursing State of the Science Initiative established a blueprint to improve genomic nursing education through development of infrastructure and research to guide genomic healthcare [89]. More recently, the Global Genomics Nursing Alliance (G2NA) was established in 2017 to focus genomic education and advancement in the general nursing population [58]. Their efforts focus on improving teaching resource accessibility and increasing collaboration between nurses around the world [86]. These efforts mark the beginning of increasing genomic competency throughout the nursing profession.

### 3.2. Genomic Education for Practicing Physicians

Physicians are often the first healthcare professional with whom patients want to discuss genetic test results; however, many practicing physicians who have not been trained in genomics in medical school do not feel prepared to use or interpret genetic test results [90,91]. It has been shown that physicians are more likely to utilize genomic technologies if they have institutional guidelines, patient interest, and clinical validity, but most importantly the knowledge and confidence needed to successfully administer tests and understand results [92]. In response to this need for genomic education for physicians, many options have become available to begin filling gaps in physician knowledge. Two major organizations have been formed to support genomic education for physicians, The Inter-Society Coordinating Committee for Practitioner Education in Genomics (ISCC-PEG) and the Genetics in Primary Care Institute (GPCI).

**Figure 4:**
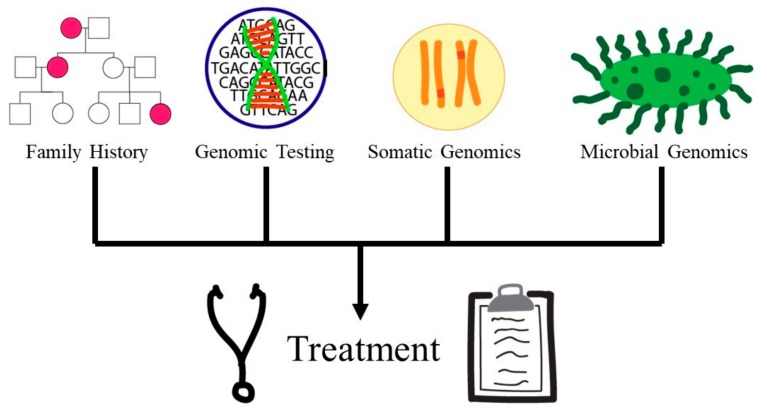
Five “entrustable professional activities” or EPAs necessary for physician instrumentation of genomics in the medical field and areas of focus for analyzing physician genomic competency adapted from [93]. These EPAs include family history, genomic testing, somatic genomics, and microbial genomics which all play a vital role in improving patient treatment.

The ISCC-PEG was formed in 2013 and designed to be a collaborative network in which physicians can address educational gaps to subsequently improve genomic knowledge and increase the use of genomics in practice [93]. The ISCC-PEG also provided a framework with which physicians can use to critically analyze genomic competency called “entrustable professional activities” or EPAs (Figure 4). Each EPA outlines principles which can guide the physician’s learning so they can understand genomics and execute these principles [94]. These principles can provide a critical foundation of genomics knowledge for physicians to utilize genomic technologies. The GCPI was established in 2011 as an electronic resource aimed at increasing genomic education and knowledge among physicians [72]. The website provides webinars on various genomics topics and resources for educating physicians. Together, these two organizations provide significant resources to aid physician genomic education and incorporation of genomic technologies into mainstream medicine.

Outside of these two organizations, other resources and models for physician genomic education are available. A recent study revealed that active learning modules used in training physicians not only improved genomic knowledge and confidence, but also increased retention of principles and subsequent changes in physician practice [95]. Educators in this study also utilized principles of backward design to identify gaps in genomic knowledge and structured the training to test genomic competency in those areas, which helped improve the educational outcomes [96]. Aside from in-person training, many other resources sites, data repositories, interprofessional education, and even emails are being used to disseminate genomic information to physicians, indicating that incorporation of genomic education into medical practice is becoming a reality [97,98].

### 3.3. Public Education

The public is perhaps one of the most difficult demographics to educate about genomics due to varying backgrounds, learning capacities, and attitudes about science [50]. However, educating the public about genomics is critical because genomics is becoming more mainstream and affects their personal lives; therefore, they need genomics education in order to make educated decisions about healthcare for themselves and their families [80,99]. Currently, many obstacles make educating the public about genomics challenging, including the media and educational infrastructure [50].

There are many websites dedicated to providing foundational genomic understanding, such as Genetic Alliance, Scitable, and the PHG Foundation [100]. The media can be a useful tool for researching genomics; however, like many other science topics, the internet also provides misleading or unhelpful information [43]. Often, people find information about genomics through news headlines, which presents a dilemma primarily centered on journalism [45]. News journalists are trained to write very differently from scientists as they generate engaging headlines or article introductions that often lack the context needed to accurately understand the genomic issue at hand [100]. Additionally, the audience may not finish reading or only skim through the article, which can perpetuate confusion and misconceptions about genomic studies [101]. To combat this challenge, more training of science policy in journalism is needed to provide thorough coverage and discussion about scientific topics, thereby providing more complete information to the public [102].

Educational infrastructure is another main concern of public genomic education. In a recent study, genomic researchers and advisors were interviewed about educating the public and found that much debate revolves around who is responsible for teaching the public about genomics, who the target audience is, and how the subject should be approached [50]. This study also revealed that there is a need for some entity to lead and centralize public genomic education, as well as develop an educational framework with which to educate the public. If a centralized organization can address these challenges, public genomic education can become more effective and beneficially help the public make decisions about their healthcare regarding genomics.

## 4. Benefits, Challenges, and Potential Strategies of Genomic Education

In this review, we have discussed genomic education on academic, professional, and public platforms. There are substantial benefits and challenges in implementing genomic education in all these areas (Table 2).

**Figure 5:**
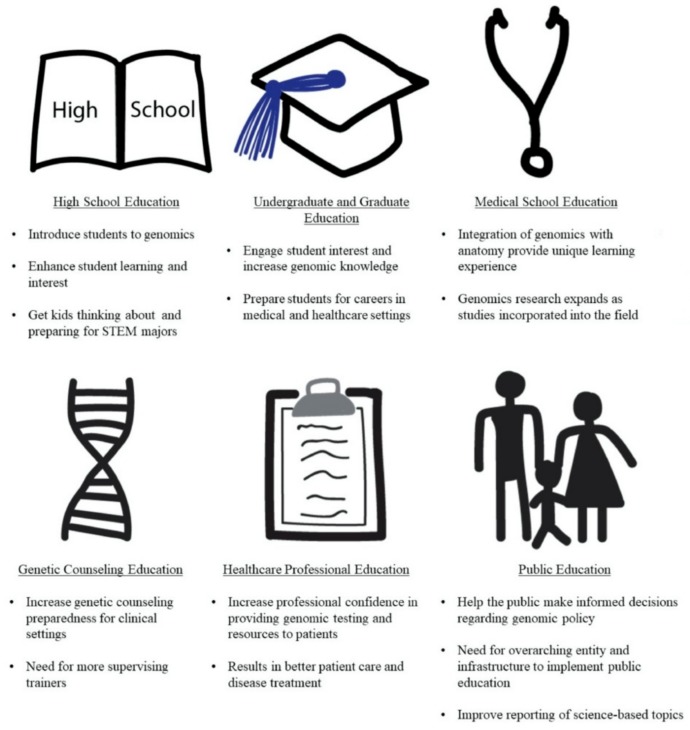
Summary of the importance of and needs for genomic education across various academic, professional, and public platforms.

As previously described, there are many benefits that genomics brings to educating students at any academic level. Numerous studies support the finding that genomic education facilitates student interest and develops student confidence [52,103]. Genomic education can also foster research ideas that increase our understanding about genomics and recruit more students to study science and prepare for scientific careers [37,77]. Studies have also shown that genomics can be scaled to teach many students and still provide meaningful learning experiences [49]. It is also important to note that the personal aspect of genomics can improve interest and engagement of learning by increasing personal investment in the learning material [46,47]. However, some challenges and barriers remain that must be addressed (Table 2). Cost and time commitments are often a challenge in providing meaningful genomic education experiences [79]. Healthcare professionals often have gaps in genomic knowledge, which can be neglected due to competing priorities and time constraints [92]. Lack of educational foundation and limited resources can hinder genomic education in different sectors [50]. Genomics complexity and misconceptions perpetuated by society or the media can compound genomic education in the public space [100]. Most importantly, genomics is rapidly expanding with new information being learned each day. Thus, it is critical to improve genomic education at the academic, professional, and public platforms to reach as many people as possible and to realize the benefits of genomics discoveries in understanding our ancestry, health, and traits (Figure 5) [29,30,31].

Despite the benefits and challenges of genomic education, it is critically important that educational dissemination and teaching strategies be addressed. Many educational approaches have been presented in this review, and here we summarize some educational guidelines which have been shown to improve genomic learning across various platforms as well as propose suggestions to address genomic education obstacles (Table 3).

Many of these strategies and suggestions can easily be applied across multiple platforms; for example, many academic platforms can incorporate personal learning experiences by having students acquire and analyze their own genomic data [46,47]. Hands-on learning experiences that utilize genomic research are also a useful tool that can be utilized for many platforms [41,68,77]. Backward education design is also a helpful education strategy to fill in existing gaps in genomic knowledge and enhance retention and learning [96]. Many easily accessible online tools, such as the Apple Genomic Project or the Genomic Analogy Model for Educators (GAME), are of valuable educational benefit in designing genomic educational strategies [40]. Together, this demonstrates that there are many educational tools and strategies that can enhance and promote learning of genomic principles.

We also have a few suggestions that educators, companies, and governments could utilize to promote genomic education. We recommend that the incorporation of genomics education be included in basic biology classes at the high school and college level in connection with human and medical genetics, since genomics is dependent upon a strong genetic understanding [33]. In regard to medical, graduate, and genetic counseling education, we suggest that educational institutions consider requiring applying students to have taken a genomics-focused or genomics-related course in order to provide a foundational genomic knowledge for incoming students. Genetic counseling programs currently struggle with providing training slots and supervision, therefore, we suggest that funding from either external or internal sources be considered to alleviate the strain on genetic counseling educators [79]. Physicians and healthcare professionals have competing interests and time constraints, so we suggest that genomic education material become more readily accessible through online formats such as email or computer-based modules in order to facilitate continuing genomic education for practicing healthcare providers [97,98]. Education for the public requires the most extensive efforts in providing accessible and pertinent genomic information [50]. As many people have suggested the need for public infrastructure and leadership over genomic education, we suggest that a public organization such as the Global Genomics Nursing Alliance (G2NA) be organized by a government entity such as the Science Education Division of the National Institute of Health to fill these educational gaps [58]. We also suggest that, with the increased use of direct-to-consumer genetic testing, these companies be required to provide online educational modules to increase public genomic education and improve public healthcare decisions [99]. In conclusion, these suggestions should provide avenues for multiple platforms for integrating and strengthening genomic education at academic and non-academic levels.

## 5. Conclusions

Effective genomics education is vital to advance our understanding of the rapidly developing field of genomics that influences our understanding of health, ancestry, and traits. Despite the challenges and barriers in implementing genomic education, there are vast benefits for academic, professional, and public education (Figure 4). Effective genomic education would not only advance genomic research, but also provide enhanced learning experiences, knowledge, and confidence for students. It is also evident that genomic education of teachers, physicians, and health care professionals should be a priority as it improves their knowledge, confidence, and ability to help communicate critical genomic information simply and accurately to students, patients, and the public. As genomics becomes more widespread and commonplace in society, genomics education becomes increasingly vital for genomic technologies to be utilized appropriately.

## Figures and Tables

**Table 1:** Publications in the last five years on high school students and genomics education.

Paper Title	Year	Learning Model	Key Finding (s)
Mining the Genome: Using Bioinformatics Tools in the Classroom to Support Student Discovery of Genes [36]	2018	NCBI and Microscope (MaGe) to map genomes	Development of effective class design
* Introducing High School Students to the Gene Ontology Classification System [37]	2018	Database of Annotation, Visualization, and Integrated Discovery (DAVID)	Prepared students for future Material enjoyable
* Spitting for Science: Danish High School Students Commit to a Large-Scale Self-Reported Genetic Study [38]	2016	Student DNA collection to present genomics	No need for costly class Positive media coverage
* Using Next-Generation Sequencing to Explore Genetics and Race in the High School Classroom [39]	2017	Next-generation sequencing and analysis by students	Learning gains, especially for lower testing students Engaged student interest
* Exploring the Effects of Active Learning on High School Students’ Outcomes and Teachers’ Perceptions of Biotechnology and Genetics Instruction [40]	2015	Apple Genomics Project Active vs. passive learning environment	Active learning had more positive Increase in knowledge No difference in interest
* Teaching the Genome Generation: Bringing Modern Human Genetics into the Classroom Through Teacher Professional Development [41]	2018	Education of teachers via TtGG program	Teacher ability increased More teaching confidence
Teaching the Big Ideas of Biology with Operon Models [42]	2015	Bacterial operons	Learn complex systems and abstract thinking Application to other academic areas
* Frame Analysis in Science Education: A Classroom Activity for Promoting Media Literacy and Learning about Genetic Causation [43]	2014	Information framing techniques	Critical thinking developed toward media
* Knowledge of, and Attitudes towards Health-Related Biotechnology Applications amongst Australian Year 10 High School Students [44]	2016	Assessment before and after genomics education	Positive attitudes developed Easy model implementation and student assessment

* indicates studies which surveyed student learning.

**Table 2:** Benefits and challenges in incorporating genomic education into academic, professional, and public settings.

Benefits of Genomic Education	Challenges of Genomic Education
Improves knowledge, interest, and engagement	Gap in knowledge among healthcare professionals
Creates positive learning that can be scaled to many	Cost, time commitment, competing priorities
Drives and strengthens genomic research	Complexity of subject material
Allows personal investment to drive learning	Misconceptions from media
Increases retainment of STEM college majors and enhances career skills and capabilities	Genomic science still developing, making implementation challenging
Develops confidence in knowledge of and communication skills about genomics	Lack of infrastructure or resources for professional development

**Table 3:** Strategies and suggestions for genomics education in academia, professional, and public settings.

Educational Platform	Strategies and Suggestions
High school	Incorporate basic principles of genomics into genetics or general biology lectures using tools such as The Apple Genomics Project or Genomic Analogy Model for Educators (GAME) [40]. Incorporate current genomics technology and tools, such as NCBI [36]. Provide active learning environment through online modules or lab-based exercises with limited teacher leading [40].
Undergraduate and graduate school	Provide personal learning experiences by incorporating personal genomic data and analysis into the classroom [46,47]. Incorporate research-based learning experiences through CUREs such as the GEP or SEA-PHAGES [66,67,68].
Medical school	Integrate genomics into genetics, anatomy, or other medical courses [76]. Institutions could require applying students to take genomics or related course before admission
Genetic counseling program	Create clinical, laboratory, or research-based rotations and hands-on experience [81,83]. Promote funding from external or internal sources to create and provide training slots and training supervision [79].
Professional development	Use backward design to fill in knowledge gaps of practicing physicians and nurses [96]. Provide materials in easily accessible formats such as online modules or emailing options [97,98].
Public education	To address lack of infrastructure and overarching entity, form an organization like Global Genomics Nursing Alliance (G2NA) run by a science education division of the National Institute of Health or other governmental entity to promote public genomic education [58]. This government agency could require that direct-to-consumer companies provide online educational modules to increase public education

## Abbreviations

GINA	Genetic Information Nondiscrimination Act
GWAS	Genome-wide association studies
TtGG	Teaching the Genome Generation
CUREs	Course-based undergraduate research experiences
GEP	Genomics Education Partnership
SEA-PHAGES	Science Education Alliance Phage Hunters Advancing Genomics and Evolutionary Science
ATG	Anatomy to Genomics
SNPs	single nucleotide polymorphisms
G2NA	Global Genomics Nursing Alliance
ISCC-PEG	Inter-Society Coordinating Committee for Practitioner Education in Genomics
GPCI	Genetics in Primary Care Institute
